# Supplementary figures and images for: Distinct metastatic organotropism shapes prognosis in lung adenocarcinoma with brain metastasis
Source: Front Oncol. 2025 Apr 4;15:1569517. doi: 10.3389/fonc.2025.1569517 (PMC12031661; doi:10.3389/fonc.2025.1569517)

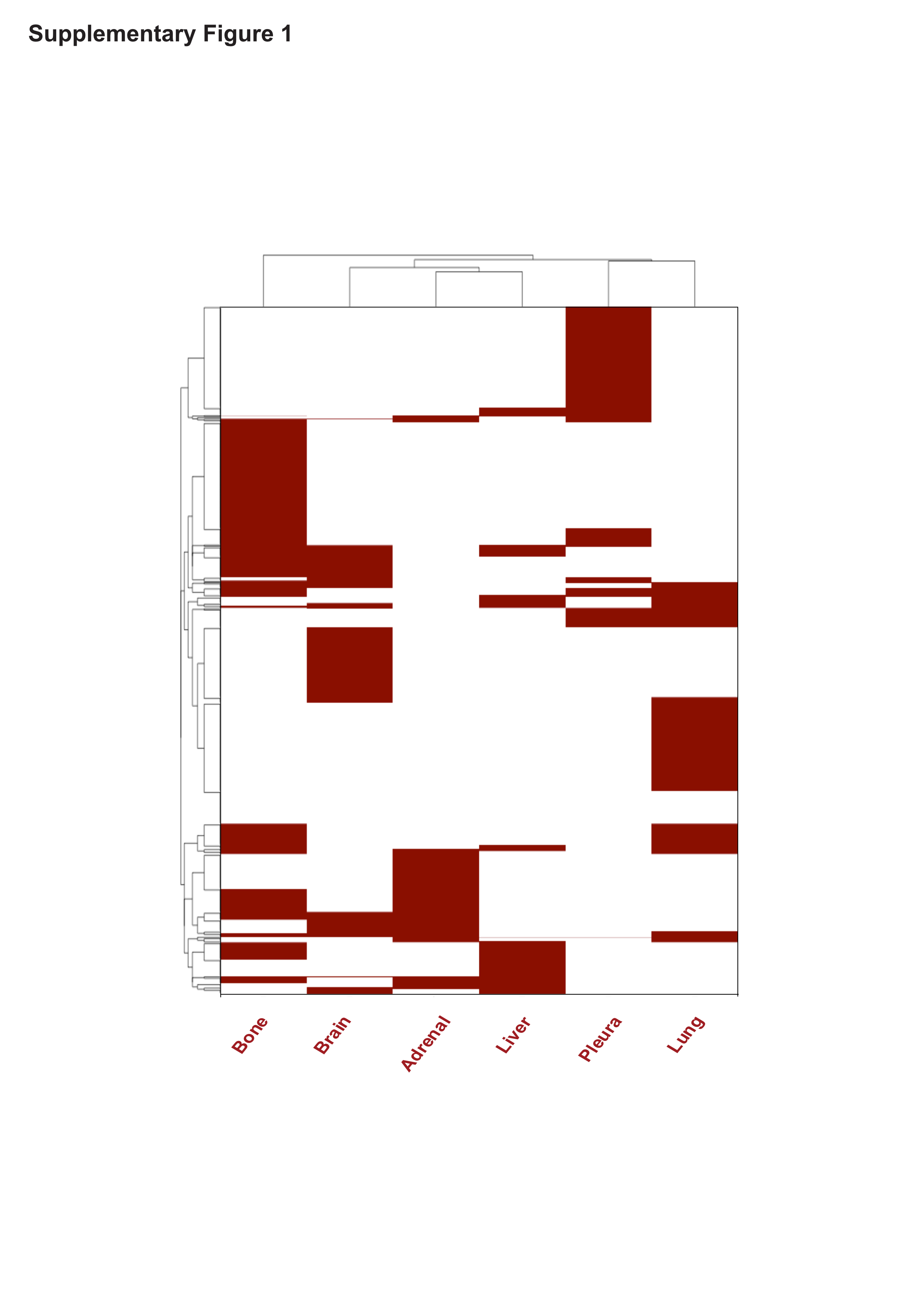

Supplement: Supplementary Figure 1 — Distribution of most frequent sites of metastasis in stage IV LUAD. Heatmap showing unsupervised hierarchical clustering of sites of metastasis in the entire study population (n = 913). Rows represent individual patients. [file Image1.jpeg]

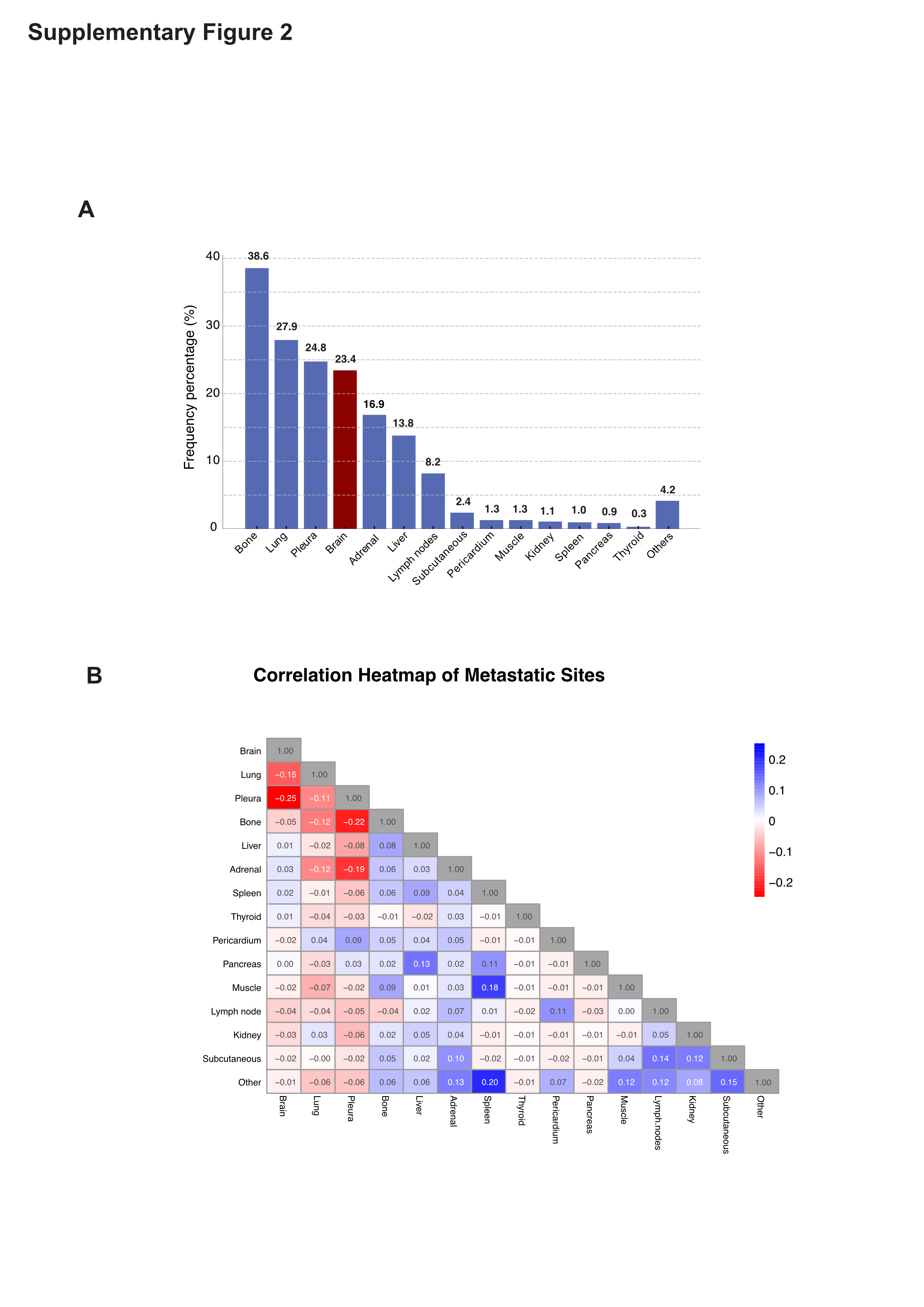

Supplement: Supplementary Figure 2 — (A) Distribution of all sites of metastasis in Stage IV LUAD, including less frequent sites (B) Pearson chart showing correlation coefficient between all organ sites of metastasis. [file Image2.jpeg]
